# How business environment shapes urban tourism industry development? Configuration effects based on NCA and fsQCA

**DOI:** 10.3389/fpsyg.2022.947794

**Published:** 2022-08-03

**Authors:** Hui Zhang, Shujing Long

**Affiliations:** The College of Tourism, Huaqiao University, Quanzhou, China

**Keywords:** business environment, tourism industry development, fsQCA, NCA, configuration effects

## Abstract

The optimization of the business environment helps to create a good market ecological environment and promote industrial development. Based on the theory of institutional complexity, this study constructs the evaluation index system of China's urban business environment and analyzes the influencing factors using the NCA method. It is found that there is no necessary condition for a single element to constitute the high-level development of the tourism industry, but improving public service, total market volume, and innovation environment play universal roles in promoting the high-level development of the tourism industry. Using fsQCA for configuration analysis, two business environment configuration paths with high-level development of the tourism industry are obtained, which shows that there is no single path to promote the development of the tourism industry. In addition, the antecedent paths of high- and low-level tourism industry development are not the opposite, presenting an asymmetric causal relationship. The above findings reveal the realization path of the business environment for the development of China's urban tourism industry. Under the logic of multiple systems, cities can find the business ecological environment that best matches the development of the local tourism industry.

## Introduction

In recent years, China has accelerated the construction of a modern economic system, and the business environment has become the main starting point to promote high-quality economic development. In 2019 and 2020, China released policies and regulations, such as Regulations on Optimizing the Business Environment and Implementing Opinions of the General Office of the State Council on Further Optimizing Business Environment and Better Serving Market Players, which shows that China is paying more and more attention to the development and optimization of the business environment. With the continuous advancement of the reform of “deregulation, management, and service”, China continues to build an international, market-oriented, and legal business environment, and the tertiary industry and emerging industries are growing rapidly. According to the global ranking of business environment measured by the world bank, China's business environment rose from 78th to 46th in 2019 and reached 31st in 2020, ranking among the top 10 economies with the largest improvement in the global business environment for 2 consecutive years. A good business environment is conducive to the development of market players and is an important aspect of highlighting the development of the competitiveness of the country or region (Nam and Tram, 2019). The institutional environment has uncertainty, heterogeneity, geographical diversity, and internal contradictions. Different institutional logics interact and blend to form different configurations, resulting in the problem of institutional complexity (Luo, 2015). In addition, the major changes in China's institutional environment have undergone major changes in the transition period, thus creating an environmental basis for analyzing the high-quality development of the tourism industry from the perspective of institutional complexity (Hitt and Xu, 2016). The business environment ecosystem has a collection of multiple factors. Identifying the core conditions, peripheral conditions, and combination paths affecting industrial development is conducive to each region to choose the high-quality development path for the tourism industry (Du et al., 2020). The development of the regional business environment and tourism economy depends on and interact with each other. The optimization of the business environment can provide institutional support for the development of the tourism industry, improve tourism-related infrastructure, and standardize tourism services (Dou, 2021). The existing literature on the impact of business environment on industrial development mainly adopts multiple regression analysis, which has great limitations in dealing with triple and above relationships. QCA breaks through the limitations of traditional regression statistics and explains the relationship between multiple repetitions and miscellaneous through the balance of case-oriented and variable-oriented and the configuration analysis of antecedent conditions (Kan et al., 2016). Based on the theory of institutional complexity, combined with the methods of fsQCA and NCA, this study takes urban tourism income as the proxy index of the development level of the tourism industry, and collects the data of 132 prefecture-level and above cities in China in 2019 from seven aspects: government environment, human resource, financial service, public service, total market volume, innovation environment, and digital economy, and analyzes how different business environment indicators affect the development of tourism industry. This study attempts to solve the following key problems: in the complex institutional environment, the convergent system is not necessarily conducive to the development of each city, so which business environment elements are the necessary conditions to promote the development of the urban tourism industry? What kind of business environment configuration can be formed by coupling urban business environment elements? Which cities' business environment configuration can better promote the development of tourism industry? The theoretical and practical contributions of this study are as follows: first, based on the theory of institutional complexity, by integrating the elements of the business environment, this study explores how the benign coupling between different systems can promote the development of the tourism industry in different types of cities and provide new institutional ideas for the development of tourism industry. Second, comprehensive fsQCA and NCA methods complement each other, give full play to their respective advantages in exploring complex causal problems, and explore whether and to what extent a single business environment element can become a necessary condition for promoting the development of tourism industry; Finally, taking cities at prefecture-level and above as research samples, this study introduces the perspective of institutional configuration, which makes the research more systematic and specific. Under the multi-institutional logic, cities can find a business ecological environment in line with the development of the local tourism industry, which has important theoretical and practical significance for promoting the development of the tourism industry.

The rest of this study is arranged as follows: Section Research methods is relevant literature on the business environment and tourism industry; Section Results analysis is the research design, including methodology, research samples, data sources, and variables; Section Further discussions is the results analysis of the path of a business environment promoting the development of tourism industry; Section Conclusion and policy implications summarizes the conclusions and policy implications.

## Literature review

After institutional isomorphism (Meyer and Rowan, 1977) and new institutionalism (Yang, 2003), institutional logic theory has developed into a new research perspective of institutional theory. The heterogeneity, dynamics, and historical variability of Institutional Logic jointly affect the complexity of the system. In the face of different institutional situations and conditions, organizations do not blindly accept, but filter out the interference and incompatible field logic, so that a variety of institutional logic in the same field can be presented differently in different organizations (Li and Liu, 2015). The business environment is the combination of multiple systems. It is the comprehensive impact of the external environment on the market subject in the whole development process. It mainly includes government affairs, finance, services, innovation, talents, market, and other factors. Currently, the research on the business environment mainly focuses on the construction and optimization of the evaluation system and the impact effect of the business environment. First, the construction and optimization of the evaluation system. Li et al. (2019) constructed the evaluation indicators of the urban business environment from the six aspects of government, human resources, finance, services, market, and innovation, measured and scored the prefecture-level and above cities in China, and put forward policy suggestions to optimize the urban business environment. Obisesan and Olayide (2020) found that business indicators such as regulations, taxes, finance, and infrastructure have become the main factors restricting the development of women entrepreneurs, and the discovery of core obstacles is conducive to helping women entrepreneurs develop better in Nigeria. Liang et al. (2021) established an evaluation model of China's business environment using the entropy weight TOPSIS method. The study found that China's urban business environment is affected by infrastructure, economic development, urban volume, and other factors, and there is an imbalance in regional development. Sul et al. (2020) constructed the tourism competitiveness model of the business environment, clarified the important dimensions and competitive advantages of the business environment of tourism destinations, and provided an important theoretical basis for tourism management and decision-making. Dvorsky et al. (2020) used the business environment data of 258 companies as a sample and found that the macroeconomic environment has a significant impact on the quality of the business environment, while the quality of the business environment in the service sector is not affected by monetary policy, corporate financing, and the consumer population. Second, it influences the effect on the business environment. Liu and Hu (2020) established an extended gravity model and confirmed that optimizing the business environment can promote the import and export bilateral trade of countries along the “Belt and Road”. Clarke et al. (2016) explored the relationship between the business environment, economic agglomeration, and employment, and found that the places where the economy is concentrated are conducive to employment growth, but there is no direct relationship between many macro business environments and employment, and the business environment factors in the labor market are more conducive to employment growth. Du et al. (2020) explored various configurations that affect high entrepreneurial activity in cities, and pointed out the importance of the government's “helping hand” to high entrepreneurial activity. The business environment has a great impact on entrepreneurial activities, thus driving regional economic development. The greater the degree of fiscal decentralization, the better the business environment. This situation is more obvious in low-income countries. Amiri and Beiranvand (2020) used panel data from 23 countries from 2010 to 2017; they analyzed business indices, good governance indices, foreign direct investment, fixed capital, government spending, and population growth, and the findings showed that business indices had a significant impact on strengthening the economy. Therefore, to strengthen the country's economic development, it is necessary to improve the business environment and strengthen labor and innovation inputs. However, under the same business environment conditions, multinational corporations usually choose countries with low entry–exit efficiency in exchange for stronger contract execution. A mature and competitive enterprise needs to be supported by a better innovative business environment (Rocha, 2012). The optimization of the business environment system is conducive to the construction of a good policy environment for enterprises, provides sufficient development confidence for enterprises, standardizes the development of market subjects, improves the vitality of market economic development, and plays a good role in promoting the development of market subjects. Different institutional logics lead to different results under different conditions, so the choice of institutional process is particularly important.

Currently, a small amount of literature has paid attention to the research on the relationship between the business environment and the tourism industry. Rigelský et al. (2021) conducted panel regression processing using the data of 36 OECD countries in the world tourism and Tourism Council database. The research found that institutional innovation had the greatest impact on Tourism expenditure. Currently, a small amount of literature has paid attention to the research on the relationship between business environment and tourism industry. Rigelský et al. (2021) empirically found that institutional innovation has the greatest impact on tourism expenditure by using the data of 36 OECD countries in the WTTC database. Gani and Clemes (2021) analyzed the tourism data of 24 countries within the framework of the gravity model and concluded that an open trade environment and a good business environment could attract more international business tourists and obtain additional benefits. Kim et al. (2018) verified that the improvement of system quality could obtain higher international tourist income, and the quality of regulation and rule of law has greatly affected the flow of international tourism. Litavcova and Vasanicova (2019) used the tourism competitiveness index released by the world economic forum to discuss the relationship between tourism competitiveness and the business environment. There is a strong correlation between them, among which human resources and the labor market have the most stable impact on tourism competitiveness. Through factor analysis, Vasanicova et al. (2021) found that the legislative system and tax are the two main business environmental factors affecting tourism competitiveness. Based on the literature review, it can be seen that some scholars have paid attention to the importance of the business environment for the development of the tourism industry, but there is no literature to explore the specific factors affecting the development of tourism industry under the complex institutional environment and the combination path under the common influence. As a factor affecting industrial development, business environment is not systematic and in-depth in the study of tourism industry development.

Based on the theory of institutional complexity, this study takes Chinese cities at the prefecture-level and above as the research object and constructs the business environment index system from the seven dimensions of government environment, human resources, financial services, public services, total market volume, innovation environment, and digital economy, and comprehensively calculates the scores of cities in each dimension. Using the method of fsQCA, this study explores the influence mechanism of multiple conditions constituting the urban business environment and the development level of the tourism industry and analyzes the influencing factors promoting the development of the tourism industry from different configurations, which has important theoretical and practical significance for exploring the multi-institutional path of promoting the development of urban tourism industry.

## Research methods

### Analytical methods

QCA was first developed by Charles C. Ragin in The Comparative Method: Moving Beyond Qualitative and Quantitative Strategies (Ragin, 1987). QCA is a relatively macro term, which is also subdivided into csQCA, mvQCA, and fsQCA in QCA technology. The core technology of QCA is Boolean algebraic logic, and the combination of conditions strongly associated with the results can be analyzed and obtained in the process of Boolean minimization (Braumoeller, 2015). Based on set theory, QCA uses multiple conditions as sets instead of more commonly used variables to express causality, which is also conducive to identifying whether antecedent conditions are necessary when analyzing causality (Gerrits and Pagliarin, 2020). This study discusses the influence path of various factors in the business environment on promoting the development of the tourism industry, that is, the influence mechanism of multiple paths on the same result. Therefore, the QCA method is used to explore the complex causal relationship between multiple interdependent antecedents and outcome predictors. The qualitative comparative analysis specifically includes three analysis methods. Crisp-set qualitative comparative analysis adopts Boolean binary logic and can only deal with binary metadata, that is, “black or white”, and use “0” and “1” to judge whether the outcome is reached (Mendel and Korjani, 2012). Compared with csQCA, multi-value QCA is more flexible. When it can divide categories for logical judgment, mvQCA is generally selected. When exploring management reality and developing new theories, csQCA and fsQCA are usually used to study management phenomena (Roig-Tierno et al., 2017). Fuzzy-set QCA introduces the concept of membership degree to set the anchor point of data. The original values of dependent and independent variables are re-processed with continuous fuzzy values. The fuzzy values are within the interval [0, 1], where 0 means completely not belonging and 1 means completely belonging, so that the data can more accurately reflect the influence brought by the changes of different degrees of antecedents conditions (Fainshmidt et al., 2020).

This study chooses to use the method of fsQCA because it can deal with partial membership problems based on Boolean algebraic logic and set analysis and can explore the multiple causality between multiple casual conditions and outcome, form an equivalent combination of multiple casual conditions, and finally achieve the effect of “the same goal through different paths” (Ragin, 2010). Compared with traditional analysis methods, QCA has four advantages. First, it is suitable for small sample sizes and can process sample size data <300 (Castro et al., 2013). Second, it means that different paths or combinations may lead to the same result. The third is causal asymmetry, that is, the occurrence and non-occurrence of outcome conditions are not necessarily opposite combinations, and different interpretation conditions may appear. Fourth, the results of regression analysis will become extremely complicated when dealing with triple or above relationships, while fsQCA breaks through the limitations of traditional regression statistics (Rasoolimanesh et al., 2021). The application of fsQCA can avoid the loss of information in the case of data membership problems, improve the accuracy of data, and more fully capture the influence of changes in antecedent conditions at different levels or degrees (Misangyi et al., 2016).

When determining whether a single variable is a necessary condition to strengthen the reliability of the data and to get a more accurate judgment, this study adds the NCA test to judge the necessary conditions of antecedents one by one. The necessary conditions presence or absence, affecting the development path of tourism with different conditions. The introduction of NCA can not only judge whether it is a necessary condition, but also know that the antecedents are necessary conditions for the outcome predictor at a specific level through the effect quantity (Dul, 2016b). This study combines the necessity condition test of fsQCA as the robustness test.

### Research samples and data sources

The city is the smallest administrative division and core carrier of the external environmental ecosystem directly faced by the tourism industry. The quality of its business environment directly affects the business activities of the tourism industry. Based on this, the study takes cities at the prefecture level and above in China as the research sample. The COVID-19 pandemic of 2020 has had a dramatic impact on tourism and other industries. After taking this into account, the study sample was selected for 2019 as it was not affected by the pandemic. The data in this study are established from the China Urban Database, China Urban–Rural Construction Statistical Yearbook, and China Regional Economic Database in the EPS data platform in 2019, as well as the Statistical Bulletin of National Economy, Social Development and Report on China's Urban Competitiveness, White Study on China's Urban Digital Economy Index and Ranking List of China's Urban Political and Business Relations. Excluding 11 cities, namely Baoding, Cangzhou, Dongying, Handan, Hengshui, Langfang, Liaocheng, Linyi, Xianyang, Yulin, and Zhaoqing, which lack a number of ordinary colleges and universities; 132 cities with complete data are taken as the research samples, which meet the requirements of the sample size required by QCA method.

### Measurement

The outcome predictor of this study is the development of the tourism industry, which is measured by the total urban tourism income. The higher the total tourism income, the higher the development level of the regional tourism industry. The selection of antecedents draws on the evaluation of China's urban business environment by Li (2021), including seven antecedents, namely government environment, human resource, financial service, public service, total market volume, innovation environment, and digital economy (for details, see Table 1).

**Table 1:** Variable list.

**Construct**	**Measurement**	**Data sources**
Government environment	Political business relations index	Ranking list of China's urban political and business relations
Human resource	Weighted average of the average wage of employees (7.6%), the number of students in Colleges and universities (47.5%), and the number of employees in the unit at the end of the year	China city database
Financial service	Weighted average of loan balance of financial institutions at the end of the year (44%) and deposit balance of financial institutions at the end of the year (56%)	China city database
Public service	Weighted average of water supply capacity (28%), gas supply capacity (48%), power supply capacity (19%), hospital beds per 10,000 people (5%)	China urban–rural construction statistical yearbook, China city database
Total market volume	Weighted average of permanent resident population (10%), per capita disposable income of urban residents (3%), import and export volume of goods (33%), regional GDP (15%), general budget income (24%) and total retail sales of social consumer goods (15%)	China regional economy database, China city database
Innovation environment	Weighted average of science expenditure (79%) and scientific and technological innovation competitiveness index (21%)	Report on China's urban competitiveness
Digital economy	Urban digital economy scale index	White study on China's urban digital economy index
Tourism industry development	Total tourism revenue	Statistical bulletin of national economy and social development

The specific measurement process of antecedents, such as government environment, human resource, financial service, public service, total market volume and innovation environment, are described in Table 1.

The sub-indicators involved in the antecedents are dimensionless. Each sub-item index involves different units and represents different meanings, which cannot be calculated and compared directly. This study uses the utility value method to deal with it dimensionless. The formula is as follows:


$$\begin{array}{l} X_{ji}=\frac{X_{ji}-minX_{ji}}{maxX_{ji}-minX_{ji}}\times 100 \end{array}$$

Where, X_*ji*_, min X_*ji*_, max X_*ji*_, and X_*ji*_ represent the original value, minimum value, maximum value, and standardized value of the sub-index of the region, respectively.

Second, the weight of each sub-index involved in the antecedents is determined. To avoid the weight deviation caused by subjective judgment, this study determines the index weight by the proportion of the variation coefficient of each sub-index in the total variation coefficient of the sub-index involved in the corresponding antecedents.

Third, the comprehensive score of antecedents is calculated. The comprehensive score of the antecedents can be obtained by weighted averaging the standardized values of each sub-index. The value of each antecedent is between 0 and 100. The closer it is to 100, the higher the score.

## Results analysis

### Variable calibration

To better meet the logic operation of Boolean algebra, it is necessary to calibrate the variables, convert the cases into sets, and determine the set membership of each variable. The methods of variable calibration are mainly divided into direct and indirect methods. This study uses the direct method and fsQCA3.0 software to calibrate the variable data. The selected qualitative anchor points refer to the upper quartile method of Du et al. (2020) and take 75% of the upper quartile, 50% of the median, and 25% of the lower quartile as the three calibration points of complete membership, intersection and complete non-membership to calibrate the antecedent and outcome variables; it describes the extent to which cases belong to a set (Ragin, 2008). The calibration value is shown in Table 2.

**Table 2:** Variable calibration value.

**Fuzzy set calibration**			
**Set**	**Full non–membership**	**Cross–over Point**	**Full membership**
Government environment	30.0225	38.17	50.2525
Human resource	4.3050	7.1414	14.1292
Financial service	1.4741	2.8469	10.1530
Public service	4.9294	7.1768	13.2847
Total market volume	3.2693	5.5807	11.3608
Innovation environment	4.0614	6.6726	11.9058
Digital economy	49.5	58.45	66
Tourism industry development	0.0679	0.1230	0.2305

### Necessity test

This study uses the NCA method in R software to test the necessary conditions (Li, 2017) of the government environment, human resources, financial service, public service, total market volume, innovation environment, and digital economy. If there is no necessary condition, the outcome cannot occur. If there is a necessary condition, the outcome is not inevitable. NCA measures the necessity of a condition by indicating at which level condition x is necessary for outcome y (Dul, 2016a). In NCA, the value of effect size (*d*) is between 0 and 1, and 0 ≤ *d* < 0.1 indicates a low level, 0.1 ≤ *d* <0.3 indicates a medium level, and 0.3 ≤ *d* < 0.5 indicates a high level (Dul, 2016b). It can be seen from the analysis results in Table 3 that except for digital economy (*P* = 1.000), other conditional variables are significant (*P* < 0.01), but because the effect size is too small, there is no business environment element as a necessary condition.

**Table 3:** NCA method test results.

**Causal condition**	**Ceiling techniques**	**Accuracy rating**	**Ceiling zone**	**Scope**	**Effect size**	***P*–value**
Government environment	CR	100%	0.002	1	0.002	0.000
	CE	100%	0.005	1	0.005	0.000
Human resource	CR	94.7%	0.040	1	0.040	0.000
	CE	100%	0.040	1	0.040	0.000
Financial service	CR	98.5%	0.018	1	0.018	0.000
	CE	100%	0.023	1	0.023	0.000
Public service	CR	99.2%	0.004	1	0.004	0.002
	CE	100%	0.005	1	0.005	0.004
Total market volume	CR	95.5%	0.038	1	0.038	0.000
	CE	100%	0.022	1	0.022	0.000
Innovation environment	CR	98.5%	0.023	1	0.023	0.000
	CE	100%	0.027	1	0.027	0.000
Digital economy	CR	100%	0.000	1	0.000	1.000
	CE	100%	0.000	1	0.000	1.000

The bottleneck level reflects the bottleneck value of the causal condition corresponding to a certain level of the outcome predictor. For example, in Table 4, to reach 60% of the tourism industry development level, there must be 2.6% level of human resource, 2.2% level of financial service, and 1.3% level of total market volume, and there is no bottleneck level in other conditions. When we want to reach 90% of the tourism industry development level, we need 1.2% level of government environment, 13.5% level of human resource, 4.3% level of financial service, 2.0% level of public service, 13.5% level of total market volume and 10.4% level of innovation environment. There is no bottleneck level in the digital economy.

**Table 4:** Bottleneck level analysis of the NCA method.

**Tourism industry development**	**Government environment**	**Human resource**	**Financial service**	**Public service**	**Total market volume**	**Innovation environment**	**Digital economy**
0	NN	NN	NN	NN	NN	NN	NN
10	NN	NN	NN	NN	NN	NN	NN
20	NN	NN	NN	NN	NN	NN	NN
30	NN	NN	NN	NN	NN	NN	NN
40	NN	NN	0.7	NN	NN	NN	NN
50	NN	NN	1.4	NN	NN	NN	NN
60	NN	2.6	2.2	NN	1.3	NN	NN
70	NN	6.2	2.9	NN	5.4	NN	NN
80	0.4	9.8	3.6	NN	9.4	4.4	NN
90	1.2	13.5	4.3	2.0	13.5	10.4	NN
100	2.0	17.1	5.1	4.2	17.5	16.8	NN

This study further uses the fsQCA method to strengthen the accuracy of necessary condition analysis. When the consistency is >0.9 (Cruz-Ros et al., 2017), it indicates that the antecedent variable is a necessary condition for the outcome variable. It can be seen from Table 5 that the consistency of all antecedent variables is <0.9, and the absence of a certain business environment element is a necessary condition, which is consistent with the results obtained by the NCA method, indicating that the results are robust.

**Table 5:** Analysis of necessary conditions of fsQCA method.

	**Outcome variable**			
**Antecedents**	**High–level development of tourism industry**		**Low–level development of tourism industry**	
	**Consistency**	**Coverage**	**Consistency**	**Coverage**
Government environment	0.756185	0.719243	0.376578	0.382187
~ Government environment	0.350454	0.345051	0.723364	0.759944
Human resource	0.789383	0.788642	0.312592	0.333229
~ Human resource	0.332603	0.311986	0.801732	0.802438
Financial service	0.797683	0.816870	0.286176	0.312700
~ Financial service	0.328844	0.301551	0.832404	0.814475
Public service	0.758691	0.751396	0.337100	0.356235
~ Public service	0.349985	0.331013	0.764749	0.771771
Total market volume	0.787191	0.782291	0.318609	0.337846
~ Total market volume	0.333699	0.314585	0.794688	0.799380
Innovation environment	0.801754	0.796515	0.296595	0.314406
~ Innovation environment	0.309897	0.292233	0.808042	0.813054
Digital economy	0.770279	0.748820	0.337540	0.350130
~ Digital economy	0.331507	0.319258	0.757852	0.778767

### Configuration analysis

This study explores the path configuration of promoting the development of the high tourism industry through the analysis of the truth table in fsQCA. First, set the threshold value of the number of cases to 1, and default the original consistency to 0.8. According to Du et al. (2020), set the PRI consistency to >0.7 and analyze the paths of three solutions: complex solution (without any logical remainder), intermediate solution (only including the logical remainder that meets the expected result conditions), and reduced solution (including all logical remainder, which will not be evaluated). In this study, the intermediate solution is the main path, and the antecedent variables that appear in both the reduced solution and the intermediate solution are the core conditions, while the antecedent variables that only appear in the intermediate solution are the peripheral conditions. As can be seen from Table 6, the overall solution consistency of high- and low-level development of the tourism industry is 0.8978 and 0.8577, respectively, which is greater than the recommended standard of full consistency of 0.8 (Fiss, 2011). The coverage of the overall solution of high-level development of the tourism industry is 0.6572, indicating that the path of high-level development of the tourism industry can explain 65.72% of the target results. The coverage of the overall solution of low-level development of the tourism industry is 0.721, indicating that the path of low-level development of tourism industry can explain 72.1% of the target results, which has a strong explanatory effect. On the whole, there is an asymmetric causal relationship between the configuration path of high-and low-level development of the tourism industry, that is, through the opposite of the influencing factors of high-level development of tourism industry, the path of low-level development of tourism industry cannot be deduced directly, which shows that the influencing factors of tourism industry development have the same goal and multiple concurrencies (Douglas et al., 2020).

**Table 6:** Configuration path of tourism industry development.

	**High–level development of tourism industry**		**Low–level development of tourism industry**						
**Antecedent condition**	**1**	**2**	**1A**	**1B**	**2A**	**2B**	**3**	**4**	**5**
Government environment	⊗		⊗	⊗		⊗	⊗	⊗	
Human resource	•	•	⊗	⊗	⊗	⊗		⊗	•
Financial service	•	•	⊗	⊗	⊗		⊗	⊗	⊗
Public service	•	•		⊗	⊗	•	⊗	⊗	⊗
Total market volume	•	•			⊗	⊗	⊗	⊗	⊗
Innovation environment	•	•	⊗	⊗	⊗	⊗	⊗		•
Digital economy		•	⊗		⊗	•	⊗	⊗	•
Consistency	0.8872	0.8988	0.8926	0.8849	0.8455	0.8835	0.8878	0.9474	0.905
Original coverage	0.1676	0.6438	0.5527	0.5147	0.5599	0.4943	0.4856	0.0926	0.0979
Unique coverage	0.0135	0.4897	0.0415	0.0148	0.0716	0.0178	0.0069	0.0075	0.0214
Overall solution consistency	0.8978		0.8577						
Overall solution coverage	0.6572		0.721						

•, •, ⊗, *and* ⊗ *indicate the presence of peripheral conditions, the presence of core conditions, the absence of peripheral conditions, and the absence of core conditions, respectively. Blank space indicates “don't care”*.

### Configuration path of high-level development of tourism industry

(1) The consistency of path 1 is 0.8872, which is greater than the acceptable level of 0.8, the original coverage is 0.1676, and the unique coverage is 0.0135. The coverage is used to compare the interpretation of results by different configurations, and there is no minimum threshold (Leppänen et al., 2019). The core conditions of high public service, high market volume, and high innovation environment combined with the peripheral conditions of high human resource, high financial service, and low government environment promote the development of the urban tourism industry, regardless of the level of the digital economy. In cities with a bad government environment, the government mainly promotes the development of the tourism industry through high public service, total market volume, and an innovation environment. Typical cities in this category include Luoyang and Tangshan. Luoyang promulgated the measures to promote the development of the private economy in 2018 and set up corresponding one-time incentive policies for the public service system, innovation and development of private enterprises, and talent introduction, which promoted the development of public services, innovation environment, and human resources. As a famous historical city, Luoyang has many cultural sites and pays more attention to the construction and improvement of infrastructure, public services, and other supporting facilities, which has laid the foundation for the development of the tourism industry. Tangshan was shortlisted as the “national demonstration city of entrepreneurship and innovation base for small and micro enterprises” in 2016, which has received national financial support and has good innovation policy assistance.
(2) The consistency of path 2 is 0.8988, which is greater than the consistency recommendation standard of 0.8. The original coverage is 0.6438, and the unique coverage is 0.4897. The core conditions of high public service, high market volume, and high innovation environment combined with the peripheral conditions of high human resource, high financial service, and high digital economy promote the development of the urban tourism industry, regardless of the level of a government environment. These type of cities includes Beijing, Chongqing, Chengdu, Suzhou, Guangzhou, Hangzhou, Wuhan, Tianjin, and other cities. For example, Chongqing issued Work Plan on Optimizing Doing Business Environment in Chongqing in 2019, which provides ideas for comprehensively standardizing government service, implementing the pilot of allowing foreign-invested travel agencies to operate outbound tourism in Chongqing, expanding the opening-up, and combining the implementation of cultural and tourism market supervision, and formed Chongqing's “negative list of market access” to clarify the objectives and responsibilities of the city's own optimization and reform. “Internet + government service” is implemented, and various work service processes are entered into the supervision platform to make the market more fair and transparent. The whole area tourism of Qiandao Lake in Hangzhou has developed well, with a large number of tourism enterprises pouring in. The reform of the business environment of “running once at most” just simplifies the business process of enterprises and greatly stimulates the vitality of market players. In innovative management, Hangzhou has optimized the yacht registration procedure according to the characteristics of existing tourism resources, which has better contributed to the development of local water tourism.

### Configuration path of low-level development of tourism industry

This study verifies the business environment with a low tourism industry development level, and there are seven configuration paths that produce low-level development of the tourism industry. Among them, 1a, 1b, 2a, and 2b form a second-order equivalent configuration due to the consistency of core conditions in the nested analysis of intermediate solution and reduced solution (Fiss, 2011). “~” stands for logical “no”, which means low-level development. Path 1 takes “~ government environment ~ financial service ~ innovation environment” as the core condition. On this basis, 1A shows that in the ecology of “~ human resource ~ digital economy”, the development level of the urban tourism industry cannot be high. 1B shows that in the ecology of “~ human resource ~ Public service”, the development level of the urban tourism industry cannot be high. Path 2 takes “~ human resources ~ total market ~ innovation environment” as the core condition. On this basis, 2A shows that in the ecology of “~ financial service ~ public service ~ digital economy”, the development level of urban tourism industry cannot be high. 2B shows that in the ecology of “~ government environment”, even if there is a “public service digital economy”, it cannot produce a high level of urban tourism industry development. Path 3 shows that in the ecology of “~ government environment ~ total market ~ innovation environment ~ digital economy ~ financial service ~ public service”, the development level of urban tourism industry cannot be high. Path 4 shows that in the ecology of “~ government environment ~ human resource ~ financial service ~ public service ~ total market ~ digital economy”, the development level of urban tourism industry cannot be high. Path 5 shows that in the ecology of “~ financial service ~ public service ~ total market”, even if there is a “human resource, innovation environment and digital economy”, it cannot promote the development of urban tourism industry.

### Robustness test

To verify whether the configuration path is robust, this study increases the original consistency from 0.8 to 0.85, it can be seen from Table 7 that the number of paths has not decreased, the presence of conditions of antecedent variables have not changed, and the consistency and coverage of the two paths have not changed greatly, indicating that the configuration test has passed and has good robustness (Zhao et al., 2020).

**Table 7:** Robustness test.

	**High–level development of tourism industry**		**Low–level development of tourism industry**						
**Antecedent condition**	**1**	**2**	**1A**	**1B**	**2A**	**2B**	**3**	**4**	**5**
Government environment	⊗		⊗	⊗		⊗	⊗	⊗	
Human resource	•	•	⊗	⊗	⊗	⊗		⊗	•
Financial service	•	•	⊗	⊗	⊗		⊗	⊗	⊗
Public service	•	•		⊗	⊗	•	⊗	⊗	⊗
Total market volume	•	•			⊗	⊗	⊗	⊗	⊗
Innovation environment	•	•	⊗	⊗	⊗	⊗	⊗		•
Digital economy		•	⊗		⊗	•	⊗	⊗	•
Consistency	0.8872	0.8988	0.8926	0.8849	0.8455	0.8835	0.8878	0.9474	0.905
Original coverage	0.1676	0.6438	0.5527	0.5147	0.5599	0.4943	0.4856	0.0926	0.0979
Unique coverage	0.0135	0.4897	0.0415	0.0148	0.0716	0.0178	0.0069	0.0075	0.0214
Overall solution consistency	0.8978		0.8577						
Overall solution coverage	0.6572		0.721						

•, •, ⊗, *and* ⊗ *indicate the presence of peripheral conditions, the presence of core conditions, the absence of peripheral conditions, and the absence of core conditions, respectively. Blank space indicates “don't care”*.

## Further discussions

This part further analyzes the typical cases. In terms of regional distribution, the proportion of cities with high-level development in the tourism industry in the eastern region, western region, central region, and northeast region is 53.85, 15.38, 19.23, and 11.54%, respectively. Among them, due to the early opening to the outside world and based on good basic conditions, the eastern region has always been in a leading position in the transformation and optimization of the business environment. In Regulations on Optimizing Business Environment issued in 2019, the State Council clearly mentioned that the eastern region should be the leading development of the business environment demonstration area, and the rest of the region should actively follow up the development. There is still a big gap between the business environment and the development of the tourism industry in the western region and Northeast China. Among them, typical cases of high-level development of the tourism industry include Beijing, Hangzhou, Tianjin, Guangzhou, Suzhou, Nanjing, and Shenzhen in the eastern region; Wuhan, Hefei, Changsha, and Zhengzhou in the central region; Chengdu, Chongqing, and Xi'an in the western region; and Dalian, Changchun, and Shenyang in the northeast region. The business environment of these cities is also in the top position. From the perspective of cities, the State Council takes Beijing, Shanghai, Chongqing, Hangzhou, Guangzhou, and Shenzhen as innovative pilot cities to optimize the business environment in China. The four municipalities directly under the central government and some provincial capital cities are typical cities to promote path 2 of high-level development of the tourism industry. That is, the core conditions of high public service, high market volume and high innovation environment combined with the peripheral conditions of high human resource, high financial service, and high digital economy promote the development of the urban tourism industry. A good business environment is a comprehensive achievement under the joint influence of the government environment, public services, total market volume, and other factors. Overall, the development of a business environment in large cities is relatively advanced, comprehensive, and coordinated. As a typical city with high-level development of the tourism industry, it also verifies that the optimization of the business environment gives better development space for tourism industry.

## Conclusion and policy implications

### Research conclusion

Based on the theory of institutional complexity, this study comprehensively uses NCA and fsQCA to analyze and draw the following conclusion: (1) through the necessity condition test, single business environment elements such as government service, human resource, financial service, public service, market aggregate, innovation environment, and digital economy are not enough to become the necessary condition to promote the development of tourism industry. (2) fsQCA analysis shows two configuration paths to promote the development of tourism industry. One is that high public service, high total market, and high innovation environment are the core conditions, complementary high human resources, high financial services and low government environment are the peripheral conditions. The other is that high public service, high total market, and high innovation environment are the core conditions, while complementary high human resources, high financial services, and high digital economy are the peripheral conditions. The existence of different configuration paths reflects that there can be multiple realization paths for the urban business environment to promote the development of the tourism industry. (3) Through the comparison of the research results, it is found that the core conditions of public service, total market, and innovation environment exist in the two paths, which shows that improving public service, total market volume, and creating an innovation environment are more important factors to promote the development of tourism industry. (4) In this study, there are two configurations of high-level development of the tourism industry and seven paths of low-level development of the tourism industry, which reflects that there is an asymmetric causal relationship between the configuration paths of different levels of tourism industry development, indicating that the influencing factors of tourism industry development have the same goal and multiple concurrencies, and the influence combination of different factors can lead to the same development results.

### Policy implications

Based on the understanding of the connotation of the business environment, this study puts forward development policy implications according to the research results to provide some reference for promoting the development of the urban tourism industry.

(1) Perfect public service and an innovation environment are conducive to promoting the development of tourism industry. First, in terms of public service, ensure the normal use and operation of scenic spots and tourism enterprises in terms of water, electricity and gas, accelerate the implementation of preferential policies and reduce the operating costs of tourism enterprises. Improve the tourism infrastructure, realize the full coverage of transportation, communication and electric power, and implement the toilet revolution. Improve the quality of public services, make use of modern technology, strengthen the construction and investment of digital economy and government services in the business environment, and create an “Internet +” model. Set up communication channels between government and tourism enterprises, actively listen to and adopt the opinions of government services, and realize the basic service guarantee in transportation, medical treatment, and other aspects. Second, create a good innovation environment and encourage mass entrepreneurship and innovation. Actively guide tourism enterprises to strengthen cooperation with other service-oriented enterprises, build a close communication platform, broaden development channels and ideas, and continuously optimize the innovative development of tourism enterprises. Increase the financial expenditure on science and technology, effectively transform and make use of scientific and technological achievements, such as the construction of smart tourism, pay attention to the patent protection system, and improve the innovative competitiveness of urban tourism.
(2) Focusing on a good government environment, public service, total market volume, and innovation environment coordinating various business environment elements to promote the better development of the tourism industry. First of all, in terms of the government environment, we should carry out innovative services and management in combination with the business problems of the local tourism industry, build tourism policies and regulations in line with their own development needs, set up special measures, and carry out characteristic, quality and efficient measures to optimize the business environment. Second, in the market, standardize the market environment and promote fair competition. Give full play to the functions of government service and supervision, strengthen the supervision during and after the event, ensure the pertinence and effectiveness of supervision, and strictly rectify the market chaos in tourism activities. Regulate the behavior of tourism market intermediaries, strengthen joint law enforcement, expand law enforcement, avoid unfair supervision or malpractice for personal gain, and maintain the order of fair competition in the tourism market. Third, simplify the access principles of tourism enterprises and actively implement innovative and efficient service measures such as “running at most once,” “one enterprise and one certificate,” and “joint handling of certificates and licenses”, so as to give birth to more tourism enterprises and operate according to market rules. Actively remove obstacles for the development of tourism enterprises, promote tourism enterprises to build high-quality tourism brands and establish a good corporate image.
(3) On the basis of the business environment, we should make up for the shortcomings of the city and promote the comprehensive and coordinated development of the tourism industry. First, in terms of human resources, increase investment in education and introduce professionals in other fields to form a diversified high-quality talent system. Encourage colleges and universities to establish links with local tourism enterprises, strengthen the combination of theory and practice of college students, and cultivate high-quality professionals. Improve human resource-related policies, including preferential policies such as talent introduction policy, talent settlement policy, and entrepreneurship subsidy policy, so as to attract and retain talents, and take a large number of young talents as reserves. Second, optimize financial services and solve the development dilemma of enterprises. Cancel operating service charges and reduce the financial burden of tourism enterprises. Improve the matching and optimization of credit database and financial credit database to maximize the efficiency of financial services. Establish a guarantee mechanism, innovate the government guarantee mechanism of small and medium-sized tourism enterprises, promote the integration of social resources, and provide financial guarantee for the development of small- and medium-sized tourism enterprises.

### Limitations and future directions

In this study, NCA and fsQCA methods are combined to ensure the robustness of the research results. However, there are inevitably some deficiencies: (1) due to the availability of data, there are some problems, such as incomplete selection of antecedent conditions and inaccurate measurement, which affect the popularization of the conclusion to a certain extent and need to be further improved in future research. (2) The advantage of fsQCA is that it can carry out Boolean logic operation through the fuzzy set and construct configuration effect analysis to achieve the same target result under different conditions, but it cannot determine the influence degree of different factors. In the future, it can be analyzed in combination with multiple regression. (3) This study uses cross-sectional data, but the business environment is a dynamic process of extension and optimization, and longitudinal tracking research can be carried out in the future.

## Data Availability Statement

The original contributions presented in the study are included in the article/supplementary material, further inquiries can be directed to the corresponding author.

## Author contributions

All authors contributed to the article and approved the submitted version.

## Funding

This work was supported by the Fujian Social Science Foundation Project Research on the Driving Mechanism and Promotion Path of High-Quality Tourism Development in Fujian Province (Grant No. FJ2021B143).

## Conflict of interest

The authors declare that the research was conducted in the absence of any commercial or financial relationships that could be construed as a potential conflict of interest.

## Publisher's note

All claims expressed in this article are solely those of the authors and do not necessarily represent those of their affiliated organizations, or those of the publisher, the editors and the reviewers. Any product that may be evaluated in this article, or claim that may be made by its manufacturer, is not guaranteed or endorsed by the publisher.
